# Literature review of vaccine-related adverse events reported from HPV vaccination in randomized controlled trials

**DOI:** 10.1186/s12610-016-0042-7

**Published:** 2016-11-21

**Authors:** Mohamed Macki, Ali A. Dabaja

**Affiliations:** 1 Department of Neurosurgery, Henry Ford Hospital, Detroit, MI USA; 2 Vattikuti Urology Institute, Henry Ford Hospital, 2799 W. Grand Blvd, Detroit, MI 48202 USA

**Keywords:** Bivalent, HPV, Human papilloma virus, Vaccine, Quadrivalent, Bivalent, HPV, Papillomavirus humain, Vaccin, Quadrivalent

## Abstract

**Background:**

The human papilloma virus (HPV) infections were addressed with two FDA-approved HPV vaccines: quadrivalent and bivalent vaccine. The objective of this manuscript is to determine the safety of the HPV vaccine.

**Results:**

A search of PubMed articles for “human papillomavirus vaccine” was used to identify all-type HPV clinical studies prior to October 2014. A refined search of clinical trials, multicenter studies, and randomized studies were screened for only randomized controlled trials comparing HPV vaccine to controls (saline placebo or aluminum derivatives). Studies were limited to the two FDA-approved vaccines. Following PRISMA guidelines, the literature review rendered 13 publications that met inclusion/ exclusion criteria. Gender was limited to females in 10 studies and males in 1 study. Two studies included both males and females. Of the 11,189 individuals in 7 publications reporting cumulative, all-type adverse events (AE), the AE incidence of 76.52 % (*n* = 4544) in the vaccinated group was statistically significantly higher than 67.57 % (*n* = 3548) in the control group (*p* < 0.001). The most common AE were injection-site reactions. On the other hand, systemic symptoms did not statistically significantly differ between the vaccination cohort (35.28 %, *n* = 3351) and the control cohort (36.14 %, *n* = 3198) (*p* = 0.223). The pregnancy/ perinatal outcomes rendered no statistically significant difference between the vaccine group and control group.

**Conclusion:**

Because the statistically significantly higher incidence of AE in the HPV vaccine group was primarily limited to injection-site reactions, the vaccinations are safe preventative measures in both males and females.

## Résumé

**Contexte:**

Les infections dues au papillomavirus humain (HPV) ont été prises en compte par deux vaccins HPV approuvés par la FDA (Food and Drug Administration: Agence américaine des Produits Alimentaires et Médicamenteux): les vaccins quadrivalent et bivalent. L’objectif de cet article est de déterminer la sécurité du vaccin HPV.

**Résultats:**

Une recherche dans PubMed des articles sur “human papillomavirus vaccine” a été utilisée pour identifier tout type d’études cliniques sur HPV antérieures à Octobre 2014. Une recherche affinée des essais cliniques, des études multicentriques et des études randomisées n’a été menée que pour les essais randomisés avec groupe témoin comparant le vaccin HPV à un témoin (solution saline comme placébo ou dérivés de l’aluminium). Seules les études utilisant les deux vaccins approuvés par la FDA ont été retenues. En suivant les recommandations PRISMA, la revue de la littérature a retrouvé 13 publications conformes aux critères d’inclusion/exclusion. Dix études impliquaient les femmes et une les hommes. Deux études incluaient à la fois des femmes et des hommes. Sur les 11 189 personnes de 7 articles rapportant tout type d’effets indésirables (EI) cumulés, l’incidence d’EI de 76,52% (n=4544) dans le groupe vacciné était significativement plus élevée que l’incidence de 67,57% dans le groupe témoin (p<0,001). Les réactions sur le site d’injection étaient l’EI le plus courant. D’autre part, les symptômes systémiques ne différaient pas significativement entre la cohorte vaccinée (35,28%; n=3351) et la cohorte témoin (36,14%; n=3198) (p=0,223). Les issues des grossesses et les issues périnatales n’étaient pas significativement différentes entre groupe vacciné et groupe placébo.

**Conclusion:**

Comme l’incidence significativement plus élevée d’EI dans le groupe vacciné se limitait principalement aux réactions sur le site d’injection, les vaccinations sont des mesures préventives sans danger pour les hommes et les femmes.

## Background

The human papillomavirus (HPV) is an important preventable cause of sexually-transmitted disease and squamous cell carcinomas. HPV types 16 and 18 have been implicated in cervical, anal, vaginal, and vulvar cancers, while types 6 and 11 cause anogenital warts. Between 2003 and 2004, the overall HPV prevalence was 26.8 % [1]. Moreover, the prevalence of HPV infections statistically significantly increased with each year of age from 14 to 24 (*p* < 0.001) [1]. The National Cancer Institute independently developed the HPV vaccine, which was subsequently sold to Merek & Co and GlaxoSmithKline for randomized controlled trials (RCTs). The HPV vaccine studies were subsequently marketed as a novel intervention to curtail the infection’s oncologic aptitude. Years of clinical trials by the pharmaceutical companies have materialized into two Food and Drug Administration (FDA)-approved HPV vaccine. First, Gardasil or Silgard (Merck & Co) is a human recombinant papillomavirus vaccine- quadrivalent types 6,11,16,18. Second, Cervarix (GlaxoSmithKline) is a bivalent human papillomavirus vaccine- types 16, 18. While the efficacy of both vaccines has been verified in randomized control studies (RCT) [2–4], the safety of these prophylactic interventions has been strongly contested in the outpatient settings.

## Methods

**Fig. 1:**
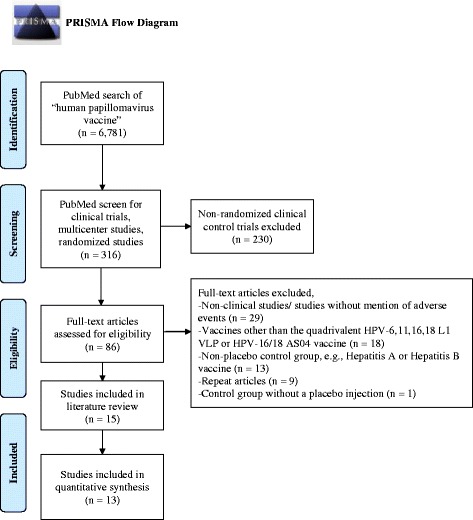
PRISMA Flow Diagram for publication selection

The literature review followed Preferred Reporting Items for Systematic Reviews and Meta-Analyses (PRISMA) guidelines (Fig. 1) [5]. A search of PubMed articles for “human papillomavirus vaccine” was used to identify all-type HPV clinical studies prior to October 2014. A refined search of clinical trials, multicenter studies, and randomized studies were screened for only randomized controlled trials comparing HPV vaccine to controls (saline placebo or aluminum derivatives). With a compilation of previously published RCTs, we compared adverse effects from the HPV vaccine versus control injection. The primary endpoint was to determine the safety of the HPV vaccine. The literature review outlined in Table 1 includes the primary author, publication year, number study participants, description of the study population, type of adverse events, number of vaccinated and unvaccinated participants for whom adverse events (AE) are reported, and *P* value comparison between the two cohorts.

**Table 1:** Literature review of vaccine-related adverse events reported from HPV vaccination in randomized controlled trials

Author, Year	Study population	Inclusion criteria	Adverse events	Vaccine group (%)	Control group (%)	*P*
Harper et al. 2004 [6]	*N* = 1113 women from 32 study sites in North America and Brazil.	15–25 years ≤6 lifetime sexual partners No abnormal Pap test No external condylomata HPV 16/18 seronegative	7-day period	*N* = 531 Gardasil	*N* = 538 Al(OH)_3_	
			Injection-site symptoms Pain Swelling Redness General symptoms Fatigue Gastrointestinal Headache Itching Rash Fever	499 (93.97) 496 (93.41) 182 (34.27) 189 (35.59) 458 (56.25) 308 (58.00) 178 (33.52) 331 (62.34) 130 (24.48) 60 (11.30) 88 (16.57)	472 (87.73) 469 (87.17) 113 (21.00) 131 (24.35) 462 (85.87) 289 (53.72) 172 (31.97) 329 (61.15) 109 (20.26) 54 (10.04) 73 (13.57)	**<0.001** **<0.001** **<0.001** **<0.001** 0.860 0.175 0.602 0.706 0.106 0.552 0.172
			Entire study period (0–27 months)			
			Vaccine-related serious adverse event Discontinuation for non-serious adverse event Discontinuation for serious adverse event^d^	0 0 1 (0.19)	0 3 (0.56) 0	1.000 0.249 0.497
Villa et al. 2005 [7]	*N* = 277 women from Brazil, Europe, and USA	16–23 years Non-pregnant No abnormal Pap smears ≤4 lifetime sex partners		*N* = 272 Gardasil	*N* = 274 AAHS	
			Vaccine-associated adverse events Injection-site Systemic Vaccine-related serious adverse events Discontinued vaccination due to hypoaesthesia	243 (89.34) 234 (86.03) 104 (38.24) 0 0	225 (82.12) 212 (77.37) 90 (32.85) 0 1 (0.36)	**0.016** **0.009** 0.188 1.000 0.319
Reisinger et al. 2007 [16]	*N* = 1781 children from 47 sites in 10 countries in North America, Latin America, Europe, and Asia, enrolled from December 2002 to September 2004	9–15 years old Sexually naïve Intact uterus No gross purulent cervicitis No genital warts No abnormal Pap smear No cervical intraepithelial neoplasia ≤4 lifetime sex partners Non-pregnant	15-day period	*N* = 1165 Gardasil	*N* = 584 Saline	
			≥1 adverse events Injection-site adverse events Erythema Pain Swelling Systemic adverse events Serious vaccine-related adverse events	963 (82.66) 877 (75.28) 237 (20.34) 853 (73.22) 241 (20.68) 541 (46.44) 0	392 (97.12) 292 (50.00) 77 (13.18) 265 (45.38) 45 (7.71) 260 (44.52) 0	**<0.001** **<0.001** **<0.001** **<0.001** **<0.001** 0.448 1.000
				*N* = 1157	*N* = 579	
			Fever	1074 (92.83)	541 (93.44)	0.638
Merck V501-013 FUTURE I Study Garland et al. 2007 [2]	*N* = 5455 women at 62 study sites in 16 countries, enrolled from January 2002 to March 2003	16–24 year old Non-pregnant No history of genital warts No abnormal cervical cytology testing ≤4 lifetime sex partners Effective contraception	5-day period	*N* = 2673 Gardasil	*N* = 2672 AAHS	
			Injection-site event Erythema Pain Pruritus Swelling	2320 (86.79) 659 (24.65) 2281 (85.33) 109 (4.70) 694 (25.96)	2068 (77.40) 450 (16.84) 2014 (75.37) 80 (2.99) 413 (15.46)	**<0.001** **<0.001** **<0.001** **<0.001** **<0.001**
			15-day period			
			Injection-related systemic event Pyrexia	1161 (43.43) 361 (13.51)	1085 (40.61) 272 (10.18)	**0.036** **<0.001**
			Entire study period			
			Vaccine-related serious event^a^ Discontinuation for vaccine-related event Death	1 (0.03) 0 2 (0.07)	0 0 2 (0.07)	0.317 1.000 0.999
Merck V501-015 FUTURE II Study, 2007 [3]	*N* = 12,167 women in 13 countries, enrolled from June 2002 to March 2003	15–26 year old Non-pregnant No abnormal Papanicolaou smear ≤4 lifetime sex partners Effective contraception	15-day period	*N* = 448 Gardasil	*N* = 447 AAHS	
			≥1 adverse event			
			Injection-site event Pain Systemic event	378 (84.38) 372 (83.04) 275 (61.38)	348 (77.85) 339 (75.84) 268 (59.96)	**0.012** **0.008** 0.662
			Entire study period	*N* = 6019	*N* = 6031	
			Serious injection-related event^b^ Discontinuation for serious injection-related event Death	3 (0.05) 0 7 (0.12)	2 (0.03) 0 5 (0.08)	0.202 1.000 0.338
Muñoz et al. 2009 [8]	*N* = 3819 women from 38 international sites in Colombia, France, Germany, Philippines, Spain, Thailand, and USA	24–45 year old Non-pregnant Intact uterus No genital warts or cervical disease HIV seronegative	15-day period	*N* = 1889 Gardasil	*N* = 1886 AAHS	
			Vaccine-related adverse events Injection-site adverse events Systemic adverse events Serious vaccine-related adverse events	1565 (82.84) 1449 (76.71) 745 (39.44) 0	1389 (73.65) 1212 (64.26) 695 (36.85) 0	**<0.001** **<0.001** 0.102 1.000
Bhatla et al. 2010 [9]	*N* = 330 women at four teaching/ tertiary care hospitals across India, enrolled from July 2006 to March 2007	Non-pregnant No investigational products/ steroids Contraception or sexual abstinence	7-day period	*N* = 171 Cervarix	*N* = 174 Al(OH)_3_	
			Pain, all-type Grade 3 Pain Redness, any size >50 mm Swelling, any size >50 mm Grade 3 solicited general symptoms Medically significant adverse event Serious adverse events Acute pancreatitis Lymph node tuberculosis Bronchogenic cyst Cataract Miscarriage Pneumothorax Death	137 (80.12) 35 (20.47) 56 (32.75) 1 (0.58) 69 (40.35) 5 (2.92) 11 (6.43) 13 (7.60) 2 (1.717) 1 (0.58) 1 (0.58) 0 0 0 0 0	105 (60.34) 7 (4.02) 24 (13.79) 1 (0.57) 35 (20.11) 3 (1.72) 10 (5.75) 24 (13.79) 4 (2.30) 0 0 1 (0.57) 1 (0.57) 1 (0.57) 1 (0.57) 0	**<0.001** **<0.001** **<0.001** 0.990 **<0.001** 0.459 0.790 0.063 0.422 0.312 0312 0.321 0.321 0.321 0.321 1.00
Ngan et al. 2010 [10]	*N* = 300 women at a single center in Hong Kong	18–35 year old No chronic disease Non-pregnant/ breastfeeding	Entire study period	*N* = 145 Cervarix	*N* = 145 Al(OH)_3_	
			Abdominal pain, IBS, dizziness, headache Pelvic inflammatory disease Medically significant conditions New onset chronic disease	3 (2.07) 0 42 (2.90) 7 (4.83)	0 1 (0.69) 24 (16.55) 5 (3.44)	0.082 0.316 **0.012** 0.555
Levin et al. 2010 [11]	*N* = 126 children HIV-seropositive children	7–12 years old HIV with CD4 ≥ 15 % ≥3 months HAART if CD4 < 25 %	14-day period	*N* = 96 Gardasil	*N* = 30 Saline Placebo	
			Adverse events Ear, eye, respiratory symptom Injection-site reactions Laboratory abnormality Systemic reactions Other	35 (36.46) 1 (1.04) 21 (21.89) 3 (3.13) 2 (2.08) 1 (1.04)	15 (50.00) 1 (3.33) 3 (10.00) 1 (3.33) 1 (3.33) 1 (3.33)	0.186 0.381 0.148 0.955 0.695 0.381
V501-20 Giuliano et al. 2011 [12, 39]	*N* = 4065 males from 71 sites in 18 countries	16–26 years old 1–5 male or female sexual partners No anogenital lesions	15-day period	*N* = 1945 Gardasil	*N* = 1950 AAHS	
			Vaccine-related events Injection-site Systemic Vaccine-related serious events Death Discontinuation for vaccine-related adverse event^e^	1242 (63.86) 1169 (60.10) 274 (14.09) 0 0 2 (0.10)	1134 (58.15) 1046 (53.64) 284 (14.56) 0 0 3 (0.15)	**<0.001** **<0.001** 0.67 1.000 1.000 0.657
			Entire study period			
			Vaccine-related events Injection-site Systematic Vaccine-related serious events Death Discontinuation for vaccine-related adverse event^e^	1242 (63.86) 1169 (60.10) 274 (14.09) 0 3 2 (0.10)	1134 (58.15) 1046 (53.64) 284 (14.56) 0 10 3 (0.15)	**<0.001** **<0.001** 0.67 1.00 0.052 0.657
Sow et al. 2013 [13]	*N* = 676 women in 2 centers in sub-Saharan Africa (Senegal and Tanzania) from October 2007 to July 2010	10–25 years old HIV seronegative Not pregnant ≤6 lifetime sexual partners	0–12 months*	*N* = 450 Cervarix	*N* = 226 Al(OH)_3_	
			Grade 3 injection-site pain Serious adverse event^c^ Medically significant condition New onset chronic disease New onset autoimmune disease Deaths Premature births- infant death	2 (0.44) 17 (3.78) 312 (69.33) 11 (2.44) 2 (0.44) 0 1 (0.22)	0 14 (6.19) 170 (75.22) 11 (4.87) 2 (0.88) 0 1 (0.44)	0.316 0.156 0.110 0.094 0.481 1.000 0.619
Yoshikawa et al. 2013 [14]	*N* = 1030 Japanese women, multicenter	18–26 years old Not pregnant No previous abnormal Pap smears ≤4 males sex partners Effective contraception	Days 1–15	*N* = 480 Gardasil	*N* = 468 AAHS	
			All-type adverse event Injection-site adverse event Systemic adverse event Serious adverse event Discontinuation for vaccine-related adverse event^f^ Death	417 (86.88) 408 (85.00) 66 (13.75) 0 1 (0.21) 0	347 (74.15) 338 (72.22) 53 (11.32) 0 0 0	**<0.001** **<0.001** 0.260 1.000 1.000
Denny et al. 2013 [15]	*N* = 150 women at a single center in Khayelitsha, Cape Town, Republic of South Africa.	18–25 years old ≤6 lifetime sexual partners Non-pregnant Intact cervix	30-day period	*N* = HIV 61 (+)/30 (-) Cervarix	*N* = 59 HIV(+) Al(OH)_3_	
			Unsolicited adverse event Headache Upper respiratory tract infection Lobar Pneumonia (Grade 3) Bacterial Pneumonia (Grade 3)	53 (86.89)/ 26 (86.67) 12 (19.67)/ 4 (13.33) 10 (16.39)/ 7 (23.33) 1 (1.64)/ 0 (0.00) 0/ 0	46 (77.97) 14 (23.73) 10 (16.95) 0 1 (1.69)	0.199 0.590 0.935/0.390 0.323/1.000 0.311/0.473
			Up to 7 months			
			Medically significant adverse event	18 (29.51)/5 (16.67)	21 (35.59)	0.477/0.063
			7–12 months	*N* = HIV54 (+)/ 24 (-)	*N* = 52 HIV(+)	
			Medically significant adverse event Discontinuation for vaccine-related adverse event	6 (11.11)/ 2 (8.33) 0/ 0	5 (9.62) 0	0.801/0.857 1.000

Statistically significant values are in bold

*Number of adverse events expressed as a function of the number of doses, rather than the number of patients, were excluded. These adverse events included malaria, headache, dysmenorrhea, abdominal pain, vertigo, cough, nasopharyngitis

Amorphous aluminum hydroxyphosphate sulfate (AAHS) adjuvant; aluminum hydroxide [Al(OH)_3_]

^a^Bronchospasm 1 day after the third dose

^b^Serious adverse events in the vaccine group were gastroenteritis, headache, hypertension, injection-site pain, and decrease in joint movement at the injection site

^c^Serious adverse events in both the vaccinated and control groups were likely due to malaria infection, unrelated to the vaccine

^d^Elective discontinuation in the vaccine group was due to spontaneous abortion, unrelated to the vaccine

^e^Elective discontinuation in the vaccine group was due to vaccine-related malaise and headache

^f^Elective discontinuation in the vaccine group was due to vaccine-related pyrexia

Both solicited and unsolicited adverse events were included in the review. AE were determined by the article investigator as possibly, probably, or definitely related to the vaccine. AE were categorized according to the discrete time intervals during which the unintended outcome occurred. HPV vaccine was typically administered in a 3-dose schedule. The number of adverse events was expressed as a proportion of subjects, rather than the proportion of doses.

### Inclusion/ Exclusion criteria

Only randomized controlled trials were included in the present article. Vaccination groups were limited to the two FDA-approved HPV vaccinations: (1) quadrivalent HPV-6,11,16,18 L1 virus-like particle (VLP) vaccine; (2) HPV-16,18 Adjuvant System (AS) 04 vaccine. Vaccines are composed of either quadrivalent or bivalent antigens plus either an amorphous aluminum hydroxyphosphate sulfate (AAHS) adjuvant or aluminum hydroxide [Al(OH)_3_]. Control cohorts were limited to solutions containing either (A) saline placebo; or (B) identical components to those in the vaccine, with the exception of the HPV antigens. Twelve articles included AAHS or Al(OH)_3_ placebo [2, 3, 6–15]; however, Reisinger et al. utilized a saline placebo [16]. RCTs with hepatitis A and/or hepatitis B vaccine controls were excluded [17–29]. Control groups without injections were also removed from the literature review insomuch as the study design would interfere with the blinded schema and become susceptible to a reporting bias/ information bias [30]. Randomized controlled trials without mention of adverse events and/or non-clinical RCTs were excluded from the literature review. Repeat studies, ad hoc subgroup analysis, and pooled analyses were similarly excluded [4, 31–38]. Lastly, AE expressed as a percentage of doses, rather than a percentage of study participants, were not included in Table 1 [13].

### Statistical analysis

Demographic information was described using summary statistics. The percent of subjects who experienced an AE in the vaccine group were compared to the placebo counterparts with Chi-squared (χ^2^) tests. Stata (version 12.0, College Station, TX, USA) and GraphPad Software were used for statistical interpretations of the raw data. Statistical significance was set a *p* ≤ 0.05.

## Results

The PRISMA flow diagram detailing the selection process is presented in Fig. 1. The most common reason for exclusion was non-randomized, clinical-controlled trials (*n* = 230). Of the 86 RCTs, the most prevalent exclusion criteria was non-clinical studies/ studies without mention of AE (*n* = 29), followed by HPV RCTs with vaccines other than the quadrivalent HPV-6,11,16,18 L1 VLP or bivalent HPV-16,18 AS04. Following PRISMA guidelines, the literature review rendered 13 publications that met the aforementioned inclusion/ exclusion criteria [2, 3, 6–16, 39]. Most clinical studies were sufficiently powered to detect a statistically significant difference between the vaccination and control cohorts, with the smallest study population of 150 women in the RCT by Denny et al. [15].

In the present literature review, the study population consisted of 31,289 subjects, 98.87 % of whom (*n* = 30,934) had follow-up data available for documentation of adverse events. Gender was limited to females in 10 studies [2, 3, 6–10, 13–15] and males in 1 study [12]. Two studies included both males and females [11, 16]. Ages ranged from 9 to 45 years. The sample populations were derived from multi-national institutions, with the exception of a Chinese trial by Ngan et al. and a Japanese trial by Yoshikawa et al. [10, 14]. Similarly, all trials were multi-center studies, with the exception of a single-institutional RCT in Hong Kong [10]. The most common study inclusion criteria were ≤4–6 lifetime sexual partners, no abnormal Papanicolaou smears, non-pregnant, and no cervical infections/ anogenital warts. Women were encouraged to utilize effective contraception. Two studies specifically focused on Human Immunodeficiency Virus (HIV)-seropositive participants [11, 15].

Of the 11,189 individuals in 7 publications reporting cumulative, all-type adverse events[7, 8, 11, 12, 14–16], the AE incidence of 76.52 % (*n* = 4544) in the vaccinated group was statistically significantly higher than 67.57 % (*n* = 3548) in the placebo group (*p* < 0.001). The most common AE were injection-site reactions. In fact, of the 18,348 participants in 9 reporting articles [2, 3, 6–8, 11, 12, 14, 16], the 77.43 % of vaccinated subjects (*n* = 7355) who experienced all-type injection-site reactions was statistically significantly higher than the 67.70 % of control subjects (*n* = 5991) (*p* < 0.001). The most common injection-site reactions were pain, induration, and erythema. On the other hand, systemic symptoms did not statistically significantly differ between the vaccination cohort (35.28 %, *n* = 3351) and the placebo cohort (36.14 %, *n* = 3198) (*p* = 0.223). The most common systemic symptoms included fatigue, headache, and fever. Ten articles (*n* = 30,398) reported serious adverse events [2, 3, 6–9, 12–14, 16]. The incidence of 0.15 % (*n* = 23) in the vaccination division did not statistically significantly differ from 0.14 % (*n* = 20) in the control counterparts (*p* = 0.774). Of the 23 subjects experiencing serious AE, 17 (73.91 %) were attributable to malaria infection, unrelated to the vaccine, in the sub-Saharan Africa study by Sow et al. [13]. Serious AE in the remaining 6 vaccinated subjects included bronchospasm, acute pancreatitis, lymph node tuberculosis, gastroenteritis, headache, and hypertension. Only 4 patients (0.03 %) in the vaccine unit [6, 12] and 7 patients (0.06 %) in the control unit [6, 7, 12, 14] discontinued the study due to adverse events (*p* = 0.367). Of the four elective terminations in the vaccine group, three men cited vaccine-related malaise, headache, and pyrexia in the publications by Giuliano et al. [12, 39] and Yoshikawa et al. [14], whereas one woman experienced a spontaneous abortion in the publication by Harper et al. [6], thought to be unrelated to the vaccine. Twelve and seventeen individuals died in the vaccine and control groups, respectively. Causes of death in the vaccine cohort included pneumonia and sepsis, overdose of an illicit drug, motor vehicle accident (6 persons), pulmonary embolism, infective thrombosis, homicide, and suicide, none of which were linked with the vaccine [2, 3, 12].

Ngan et al. and Sow et al. reported new-onset chronic disease/ autoimmune disease following injection with drug vs control [10, 13]. Of the 966 enrollees in the two studies, the rate of 3.36 % in the vaccine randomization did not statistically significantly differ from 4.85 % in the control randomization (*p* = 0.246). Lastly, while effective contraception and non-pregnancy represented key selection criteria for most RCTs in the present literature review, pregnancy was reported in the follow up period. Birth complications included one spontaneous abortion, nine elective abortions, and one death of a premature infant in the vaccination cohort in comparison to one spontaneous abortion, one miscarriage, one ectopic pregnancy, three elective abortions, and one death of a premature infant in the control cohort. None of these experiences were liked with the injections. The pregnancy/ perinatal outcomes from the Females United to Unilaterally Reduce Endo/Ectocervical Disease (FUTURE) I (Merck V501-013), FUTURE II (Merck V501-015), Merck V501-016, and Merck V501-018 RCTs were combined in the Appendix in the FUTURE II study (Merck V501-016, 018 did not meet selection criteria in this literature review) [38]. In brief, no statistically significant difference was observed between the vaccine group and control group.

## Discussion

In 1796, an English physician, Edward Jenner, performed the first vaccination by inoculating an 8-year-old boy with pus from a cowpox lesion [40]. Despite the growing safety concerns for his experimental design of a smallpox vaccine, Dr. Jenner published his conclusions in a landmark text in the annals of medicine: *Inquiry into the Causes and Effects of the Variolae Vaccine* [41]. Since the development of Dr. Jenner’s time-honored work, vaccine production over the ensuing centuries have ushered in a new era of preventive medicine; nevertheless, safety concerns for vaccination of children and young adults still remains the greatest barrier to these scientific advancements. A testament to this belief today, the most recent FDA-approved vaccines, Gardasil/Silgard and Cervarix, have met significant resistance owing to the fear of unknown side-effects. In this literature review, we compared adverse effects from the HPV vaccine versus control injection from a compilation of published randomized controlled trials. The primary endpoint of this study was to determine the safety of the HPV vaccine.

In the present literature review, the vaccine was well-tolerated without undue AE. All-type AE and injection-related AE were the only two parameters with a significantly higher rate in the HPV vaccinated subjects, whereas systemic events, serious AE, and death did not differ. The vaccine cohort (76.52 %) carried an approximately 10 % higher rate of all-type adverse events in comparison to the control cohort (67.57 %) (*p* < 0.001). These results corroborate a sub-analysis by Moreira et al. [39], who reviewed AE in the 4065 males enrolled in the HPV RCT, V501-20 published by Giuliano et al. [12]. The 1945 males randomized to the Gardasil unit experienced a statistically significantly higher rate of all-type AE (63.86 %) versus AAHS adjuvants (58.15 %) (*p* < 0.001). In fact of the 7 publications reporting all-type adverse events, 5 found a significant difference between the two cohorts [7, 8, 12, 14, 16]. The remaining two articles reporting no difference were limited by a cohort size of less than 100 persons [11, 15]. The most common AE was injection-site reactions, such as pain, erythema, and induration. In the present literature review, all-type injection-site reactions were statistically significantly higher in the vaccine arm (77.43 %) than the control arm (67.70 %) (*p* < 0.001). However, true injection-site, hypersensitivity reactions occur infrequently, according a retrospective review of 380,000 doses of Gardasil administered to 12–26 year-old females in Victoria and South Australia [42]. In that study, only 35 females had suspected hypersensitivity reactions. Moreover, Kang et al. concluded that “only three of the 25 evaluated schoolgirls had probable hypersensitivity to the quadrivalent human papillomavirus vaccine [42].” Several authors contend that the causes of the general injection-site reactions, and the hypersensitivity experiences specifically, are not completely attributable to the antigenic components of the vaccine, but rather due in part to the aluminum additives [39, 42]. In the RCT by Reisinger et al. (Table 1), the placebo group was given saline injections, from which only half of participants experienced injection-site reactions [16]. By comparison, the frequency of injection-site reactions averages at 68.95 % in control arms with Al(OH)_3_ or AAHS and 77.43 % in the vaccinated arm, per the set literature review. Such a gradient effect suggests that the aluminum products contribute to the reactogenicity of the vaccine [39].

Systemic events did not differ in the vaccine division (35.28 %) versus the control division (36.14 %) (*p* = 0.223); furthermore, most reported symptoms were mild or moderate in intensity. Fatigue, headache, and pyrexia were most commonly documented throughout the follow up period. Delayed in onset, these experiences likely reflect the initial innate immunologic response followed by a sustained, adaptive response. Yoshikawa et al. did detect a statistically significant difference of all-type adverse events between the vaccine arm and control arm (*p* < 0.001) (Table 1) [14]. The most common AE was injection-site adverse event, among which pain was the most frequent symptom. Systemic AE were the next most common event, although no statistically significant difference was detected between the vaccine and control cohorts (*p* = 0.260). Greater than 90 % of those systemic AEs were of “moderate intensity,” without any specification.

Serious AE in the present study did not statistically significantly differ between the vaccine (0.15 %) and control (0.14 %) groups (*p* = 0.774) in the present literature review. Commensurate with our findings, Roumbout et al. reported no difference in serious AE in a systematic review of six HPV trials (Peto odds ratio 1.00; 95 % CI 0.87–1.14). Death between the two arms did not differ (Peto odds ratio 0.91; 95 % CI 0.39–2.14) [43]. In the aforementioned review by Roumbout et al. as well as the present review, motor vehicle accidents were the most common cause of death. No mortalities were associated with the vaccine.

## Conclusion

Following PRISMA guidelines, the literature review rendered 13 randomized controlled trials comparing HPV vaccine to control. Of the 11,189 individuals in 7 publications reporting cumulative, all-type adverse events, the vaccinated group was statistically significantly higher than the control group, although the most common AE were injection-site reactions. On the other hand, systemic symptoms did not statistically significantly differ. The pregnancy/ perinatal outcomes rendered no statistically significant difference between the vaccine group and control group. Thus, the vaccinations are safe preventative measures for both males and females.

## Abbreviations

[Al(OH)_3_]: Aluminum hydroxide
AAHS: Amorphous aluminum hydroxyphosphate sulfate
AE: Adverse events
AS: Adjuvant System
FDA: Food and Drug Administration
FUTURE: Females United to Unilaterally Reduce Endo/Ectocervical Disease
HIV: Human Immunodeficiency Virus
HPV: Human papilloma virus
PRISMA: Preferred Reporting Items for Systematic Reviews and Meta-Analyses
RCTs: Randomized controlled trials
VLP: Virus-like particle
